# Impaired Respiratory Function as an Auxiliary Marker for Disease Progression in Different Motor Subtypes of Parkinson's Disease

**DOI:** 10.1002/mdc3.70089

**Published:** 2025-05-06

**Authors:** Zhen Li, Tinglan Huang, Ning Zhou, Xiao Shen, Yanlin Wang, Pengcheng Huang, Qingqing Shi, Tingyu Cao, Yibo Zhi, Huiran Wang, Xiaodong Zhu

**Affiliations:** ^1^ Department of Neurology Tianjin Neurological Institute, Tianjin Medical University General Hospital Tianjin China; ^2^ Department of Respiratory and Critical Care Medicine Tianjin Medical University General Hospital Tianjin China

**Keywords:** respiratory function, Parkinson's disease, motor subtype, marker, disease progression

## Abstract

**Background:**

The mechanisms underlying respiratory function impairments in different motor subtypes of Parkinson's disease (PD) remain poorly understood.

**Objectives:**

This study aims to elucidate the differences in respiratory function between the two main PD motor subtypes, tremor‐dominant (TD) and postural instability gait difficulty (PIGD), and investigate their associations with disease severity.

**Methods:**

A total of 106 PD patients (59 TD and 47 PIGD) and 42 age‐ and sex‐matched healthy controls (HCs) were enrolled. Pulmonary function tests (PFTs), respiratory muscle strength, and respiratory drive measurements were conducted. Multivariate models were employed to analyze correlations between respiratory function indices and clinical characteristics.

**Results:**

Compared to HCs, PIGD patients exhibited significant reductions in forced vital capacity (FVC%) (*P* = 0.018), peak expiratory flow (PEF%) (*P* < 0.001), diffusing capacity for carbon monoxide adjusted for hemoglobin (DLCOc%) (*P* = 0.013), and total lung capacity (TLC%) (*P* = 0.045). Although differences between the two motor subtypes were not statistically significant, a more apparent declining trend was observed in PIGD patients. Regarding respiratory muscle strength, PIGD patients showed more severe impairments in maximal inspiratory pressure (PImax%) (*P* < 0.001) and maximal expiratory pressure (PEmax%) (*P* < 0.001). Multivariate linear regression analysis confirmed that PEF% was a significant determinant of cognitive function in TD patients (β = 0.497, *P* < 0.001), whereas the airway occlusion pressure at 100 milliseconds (P0.1%) influenced cognitive levels in PIGD patients (β = −0.373, *P* = 0.015).

**Conclusions:**

Compared to TD patients, PIGD patients have more pronounced respiratory impairments. Specific respiratory indices correlate with motor severity and cognitive decline, highlighting their potential as markers for disease progression in PD motor subtypes.

Parkinson's disease (PD) is a common chronic neurodegenerative disorder characterized by pathological α‐synuclein aggregation in the central and peripheral nervous systems, accompanied by a spectrum of motor and non‐motor symptoms, which have significant implications for quality of life.^1^, ^2^ According to the established Movement Disorder Society (MDS) classification criteria,^3^ PD patients can be categorized into tremor‐dominant (TD), postural instability gait difficulty (PIGD), and indeterminate subtypes, each exhibiting distinct clinical features, therapeutic responses, imaging patterns, and progression trajectories.^4^ However, the current classification of motor subtypes relies heavily on clinical evaluation and lacks objective and reliable markers to differentiate subtypes and predict disease progression.

Emerging evidence demonstrates that respiratory dysfunction is a notable yet underrecognized feature of PD. Studies have identified alterations in maximal voluntary ventilation (MVV),^5^ lung volume,^6^ and the airway occlusion pressure at 100 milliseconds (P0.1)^7^ among PD patients. Furthermore, individuals with poor respiratory function are at increased risk of prodromal and clinical PD, with higher PD‐related mortality.^8^ Meta‐analyses have demonstrated consistent patterns of restrictive ventilatory impairment and inspiratory muscle weakness in PD, potentially serving as early markers of disease severity.^9^ Additionally, impaired respiratory function has been linked to cognitive decline in older adults, with some evidence suggesting that it may accelerate the progression of dementia.^10^ Despite these findings, the association between respiratory dysfunction and disease severity in PD, particularly in different motor subtypes, remains unclear.

Given the poorly understood characteristics of respiratory dysfunction and its potential associations with motor and cognitive impairments in PD motor subtypes, further investigation into respiratory function is warranted. Therefore, this study aims to investigate the differences in respiratory function in PD motor subtypes and to explore the relationship between respiratory function indices and disease progression. These findings are expected to clarify the changes in respiratory function and promote the development of respiratory‐based markers for disease monitoring in PD motor subtypes.

## Patients and Methods

### Participants

All PD patients were recruited from the Movement Disorders outpatient clinics at Tianjin Medical University General Hospital between April 2018 and June 2024. PD diagnoses were based on the UK Brain Bank criteria,^11^ confirmed by movement disorder specialists through clinical evaluation and imaging. PD subtypes were classified according to the MDS Unified Parkinson's Disease Rating Scale (MDS‐UPDRS) classification criteria,^3^ using the ratio of mean MDS‐UPDRS tremor scores to mean MDS‐UPDRS PIGD scores. Patients were categorized as TD (ratio ≥1.15), PIGD (ratio ≤0.90), and intermediate (0.90 < ratio <1.15). Intermediate cases were excluded from the study. Finally, a total of 59 TD and 47 PIGD patients were enrolled in our analysis. For comparisons of respiratory function across cognitive states, PD patients were stratified into normal cognition (Montreal Cognitive Assessment [MoCA] score ≥ 26), mild cognitive impairment (MoCA score 21–25), and dementia (MoCA score ≤ 20).^12^ Additionally, 42 age‐ and sex‐matched healthy controls (HCs) were recruited from the community with no significant neurological, psychiatric, or systemic diseases (especially respiratory diseases), and no family history of idiopathic PD. The study protocol was approved by the Ethics Committee of Tianjin Medical University General Hospital. Written informed consent was obtained from all participants prior to their inclusion in the research.

### Data Collection

Data collection followed the standardized protocols established at our research center. Well‐trained professionals conducted face‐to‐face interviews, clinical examinations, and neuropsychological assessments. Demographic data (eg, age, sex), lifestyle factors (eg, smoking), and medical history (eg, chronic obstructive pulmonary disease [COPD], asthma, diabetes, cardiovascular diseases) were systematically recorded. Comprehensive motor and non‐motor assessments were performed. The motor symptoms of PD patients were evaluated using the modified Hoehn and Yahr staging scale (H&Y stage)^13^ and MDS‐UPDRS Part III (MDS‐UPDRS III),^14^ administered during “off” medication, more than 12 hours after the last dose of dopaminergic therapy. Global cognitive function was assessed with MoCA. Other non‐motor symptoms were examined with a series of neuropsychological assessments, including Hamilton Depression Rating Scale‐17 (HAMD‐17), Hamilton Anxiety Rating Scale (HAMA), Parkinson's Disease Sleep Scale‐2 (PDSS‐2), Epworth Sleepiness Scale (ESS), and Cleveland Clinic Score (CCS).

### Measurements of Pulmonary Function, Respiratory Muscle Strength, and P0.1

Pulmonary function tests (PFTs) and measurements of respiratory muscle strength and P0.1 were conducted by certified technicians blinded to patient diagnoses, adhering to the standards set by the American Thoracic Society. The assessments were performed using a spirometer and a respiratory actuator (MasterScreen, JAEGER, Germany). Each subject underwent the tests in a seated position in the outpatient pulmonary function examination room of Tianjin Medical University General Hospital between 8 am and 3 pm under a controlled indoor temperature of 25–28°C. All assessments were performed during the “off” period of dopaminergic therapy.

We collected a comprehensive set of pulmonary function parameters, including spirometry, lung volumes, and diffusion capacity for carbon monoxide adjusted for hemoglobin (DLCOc). Lung volume measurements encompassed total lung capacity (TLC), residual volume (RV), and functional residual capacity (FRC). Spirometry parameters included forced vital capacity (FVC), forced expiratory volume in 1 second (FEV1), peak expiratory flow (PEF), maximum expiratory flow rate at 25% of FVC exhalation (MEF25), maximum expiratory flow at 50% of FVC (MEF50), and maximum expiratory flow at 75% of FVC (MEF75). In addition, respiratory muscle strength was assessed separately using measurements of maximal inspiratory pressure (PImax) and maximal expiratory pressure (PEmax). The P0.1 was measured to evaluate respiratory center drive. The primary outcomes were the actual measured values expressed as a percentage of the predicted values.

### Statistical Analyses

All statistical analyses were performed using GraphPad Prism version 10 (GraphPad Software Inc., La Jolla, CA, USA). Quantitative variables were presented as mean ± standard deviation (SD), whereas categorical variables were expressed as percentages. Independent *t* tests were used for two‐sample comparison. Multiple group comparisons were conducted using the Kruskal–Wallis test and analysis of covariance (ANCOVA), adjusting for potential confounders, including age, sex, smoking status, and chronic comorbidities. Bonferroni correction was applied for multiple comparisons. Spearman's rank correlation was used to evaluate the relationship between respiratory function parameters and clinical scales. The diagnostic accuracy was evaluated using the area under the receiver operating characteristic (ROC) curve (AUC). To explore the comprehensive impact of respiratory function parameters on cognitive performance, multiple linear regression models were performed with cognitive scores as the dependent variable and respiratory function parameters as predictors, controlling for age, sex, smoking, and chronic diseases. Statistical significance was set at *P* < 0.05.

## Results

### Demographic and Clinical Characteristics

A total of 106 PD patients and 42 HCs were enrolled in this study. The demographic and clinical characteristics of all participants are summarized in Table 1.

**TABLE 1:** Demographic and clinical characteristics

Variables	TD (n = 59)	PIGD (n = 47)	HCs (n = 42)	*P*‐value
Male, %	45.76	44.69	45.24	0.994
Age, y	63.81 ± 6.98	64.28 ± 7.73	62.40 ± 6.05	0.222
Disease duration, y	3.71 ± 3.41	4.60 ± 3.94	NA	0.278
Hypertension (n [%])	13 (22.03)	15 (31.91)	NA	0.275
Diabetes mellitus (n [%])	5 (8.47)	7 (14.89)	NA	0.363
COPD	NA	NA	NA	NA
Asthma	NA	NA	NA	NA
Ever smoking (n [%])	1 (1.69)	5 (10.64)	NA	0.086
Hoehn and Yahr stage	1.32 ± 0.50	1.88 ± 1.19	NA	0.008
MDS‐UPDRS Ш	20.81 ± 11.37	27.74 ± 20.25	NA	0.266
MoCA	23.98 ± 4.74	23.37 ± 4.80	NA	0.457
HAMD	5.05 ± 5.04	7.98 ± 6.06	NA	0.008
HAMA	3.83 ± 4.82	7.59 ± 8.15	NA	0.016
PDSS‐2	6.88 ± 6.92	10.63 ± 8.97	NA	0.034
CCS	5.53 ± 3.14	6.49 ± 4.24	NA	0.196
ESS	2.76 ± 3.33	3.20 ± 4.85	NA	0.925

Abbreviations: TD, tremor‐dominant patients; PIGD, postural instability gait difficulty patients; HCs, healthy controls; NA, not applicable; COPD, chronic obstructive pulmonary disease; MDS‐UPDRS Ш, Movement Disorder Society Unified Parkinson's Disease Rating Scale Part III; MoCA, Montreal Cognitive Assessment; HAMD, Hamilton Depression Rating Scale; HAMA, Hamilton Anxiety Rating Scale; PDSS‐2, Parkinson's Disease Sleep Scale‐2; CCS, Cleveland Clinic Score; ESS, Epworth Sleepiness Scale.

There were no significant differences in age or sex between PD patients and HCs. However, compared to the TD group, the PIGD group exhibited significantly higher H&Y staging, HAMA scores, HAMD scores, and PDSS‐2 scores, indicating more severe motor and non‐motor symptoms in patients with PIGD. No significant differences were observed between the two PD subgroups in terms of disease duration, history of hypertension or diabetes, smoking, MDS‐UPDRS III scores, or MoCA scores (*P* > 0.05).

### Comparison of Respiratory Function Parameters in Different Groups

The comparison of respiratory function parameters across groups is presented in Table 2. For spirometry, FVC% was significantly lower in the PIGD group than in HCs (*P* = 0.018), whereas PEF% showed a marked reduction in both PD subgroups compared to the HCs group (*P* < 0.001). Additionally, DLCO% (*P* = 0.013) and TLC% (*P* = 0.045) were notably lower in the PIGD group than in HCs.

**TABLE 2:** Comparison of respiratory function parameters in different Parkinson's disease (PD) motor subtypes and healthy controls (HCs)

Variables	TD (n = 59)	PIGD (n = 47)	HCs (n = 42)	Group differences
Lung volume				
TLC%	89.25 ± 10.40	87.50 ± 11.08	93.32 ± 11.33	*P* = 0.038^c^
RV%	91.84 ± 18.72	97.41 ± 18.92	96.00 ± 17.12	*P* = 0.207
FRC%	92.25 ± 22.26	95.97 ± 19.18	96.08 ± 16.41	*P* = 0.562
Spirometry				
FEV1%	100.90 ± 16.51	96.08 ± 15.73	103.10 ± 12.45	*P*= 0.092
FVC%	102.57 ± 16.72	97.44 ± 14.26	106.05 ± 14.01	*P* = 0.022^c^
PEF%	100.14 ± 11.37	94.47 ± 16.93	109.27 ± 13.93	*P* < 0.001^b^, ^c^
MEF25%	73.77 ± 38.39	75.13 ± 26.22	67.75 ± 33.30	*P* = 0.210
MEF50%	86.25 ± 28.57	80.50 ± 24.57	83.72 ± 23.51	*P* = 0.670
MEF75%	95.24 ± 23.44	88.82 ± 27.88	102.20 ± 19.22	*P* = 0.148
Diffusion capacity				
DLCOc%	82.34 ± 15.19	80.27 ± 12.77	88.30 ± 13.78	*P* = 0.012^c^
Respiratory muscle strength				
PImax%	37.20 ± 16.92	27.44 ± 12.65	50.15 ± 15.84	*P* < 0.001^a^, ^b^, ^c^
PEmax%	69.88 ± 24.81	55.82 ± 21.91	85.76 ± 27.68	*P* < 0.001^a^, ^b^, ^c^
Respiratory center drive				
P0.1%	156.55 ± 82.72	180.98 ± 80.66	78.22 ± 17.53	*P* < 0.001^b^, ^c^

Abbreviations: TD, tremor‐dominant; PIGD, postural instability gait difficulty; TLC%, total lung capacity; RV%, residual volume; FRC%, functional residual capacity; FEV1%, forced expiratory volume in 1 second; FVC%, forced vital capacity; PEF%, peak expiratory flow; MEF25%, maximum expiratory flow rate at 25% of FVC exhalation; MEF50%, maximum expiratory flow at 50% of FVC; MEF75%, maximum expiratory flow at 75% of FVC; DLCOc%, diffusing capacity for carbon monoxide adjusted for hemoglobin; PImax%, maximal inspiratory pressure; PEmax%, maximal expiratory pressure; P0.1%, airway occlusion pressure at 100 milliseconds.

^a^ Differences were found between TD and PIGD.

^b^ Differences were found between TD and HCs.

^c^ Differences were found between PIGD and HCs.

Regarding respiratory muscle strength, both PImax% and PEmax% showed substantial reductions in PD patients compared to HCs (*P* < 0.001 for both), with the PIGD group exhibiting lower values than the TD group. Notably, P0.1% values were elevated in PD patients compared to HCs, with no significant differences between the TD and PIGD subgroups.

### Diagnostic Accuracy of Respiratory Function Parameters for PD Motor Subtypes

**Fig. 1:**
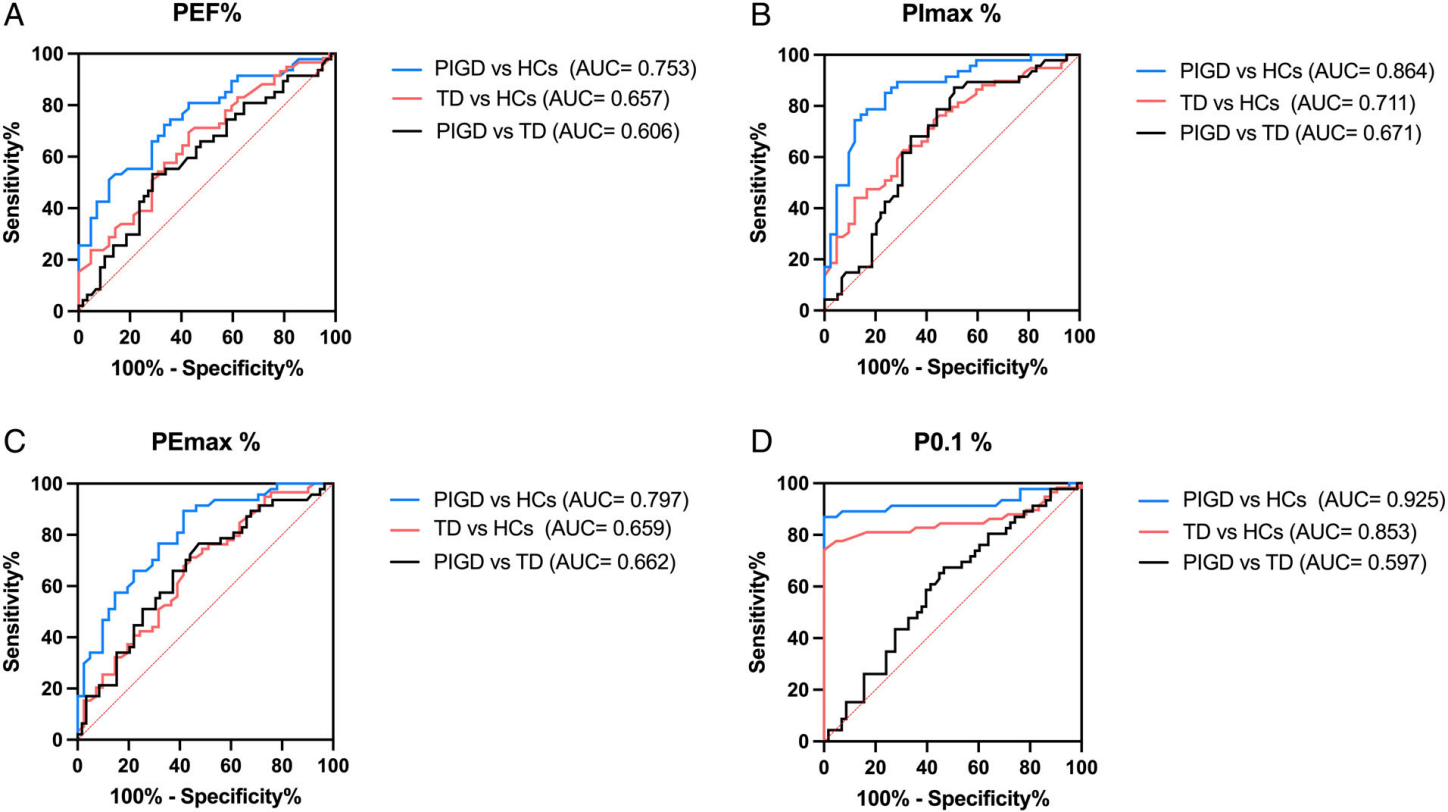
Receiver operating characteristic (ROC) curves for peak expiratory flow (PEF%) (**A**), maximal inspiratory pressure (PImax%) (**B**), maximal expiratory pressure (PEmax%) (**C**), and P0.1% (**D**) in differentiating Parkinson's disease (PD) from healthy controls (HCs) and different PD motor subtypes.

ROC curve analysis was conducted for those pulmonary function measures displaying statistically significant intergroup differences (Fig. 1). P0.1% exhibited superior diagnostic performance in distinguishing the PIGD group from HCs, with an AUC of 0.925, an optimal cutoff of 99.40%, sensitivity of 86.96%, and specificity of 100%. Similarly, P0.1% exhibited the strongest discriminative power for differentiating the TD group from HCs, with an AUC of 0.853, an optimal cutoff of 100.10%, sensitivity of 74.14%, and specificity of 100% (Fig. 1D). However, no significant advantage was observed for respiratory function parameters in differentiating motor subtypes.

### Correlation Between Respiratory Function Parameters and Clinical Symptoms

**Fig. 2:**
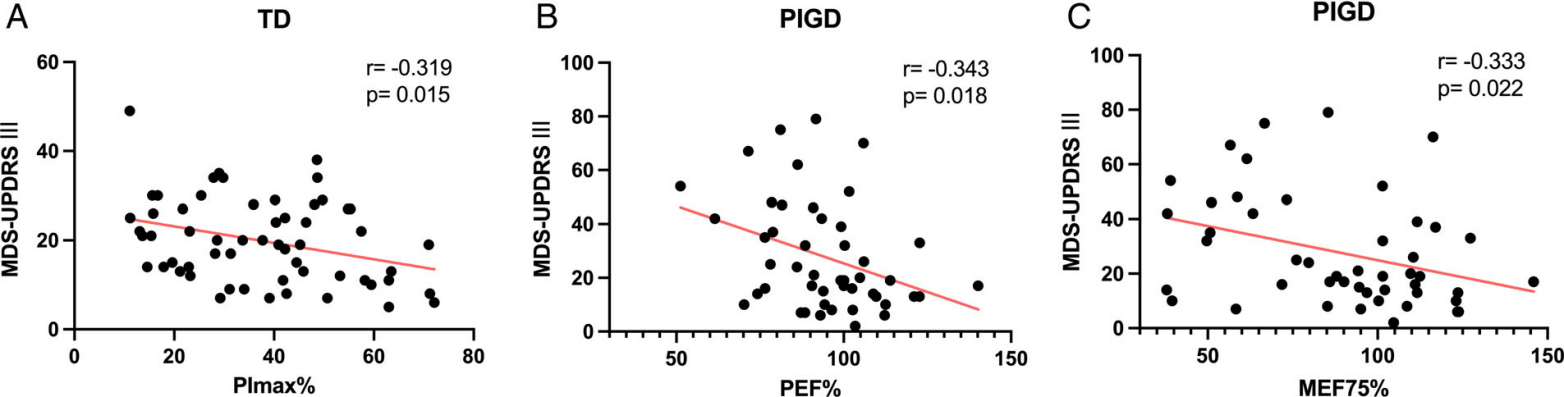
Correlation between respiratory function parameters and Movement Disorder Society Unified Parkinson's Disease Rating Scale Part III (MDS‐UPDRS III). Significant correlation (*P* < 0.05) is marked in solid red line.

**Fig. 3:**
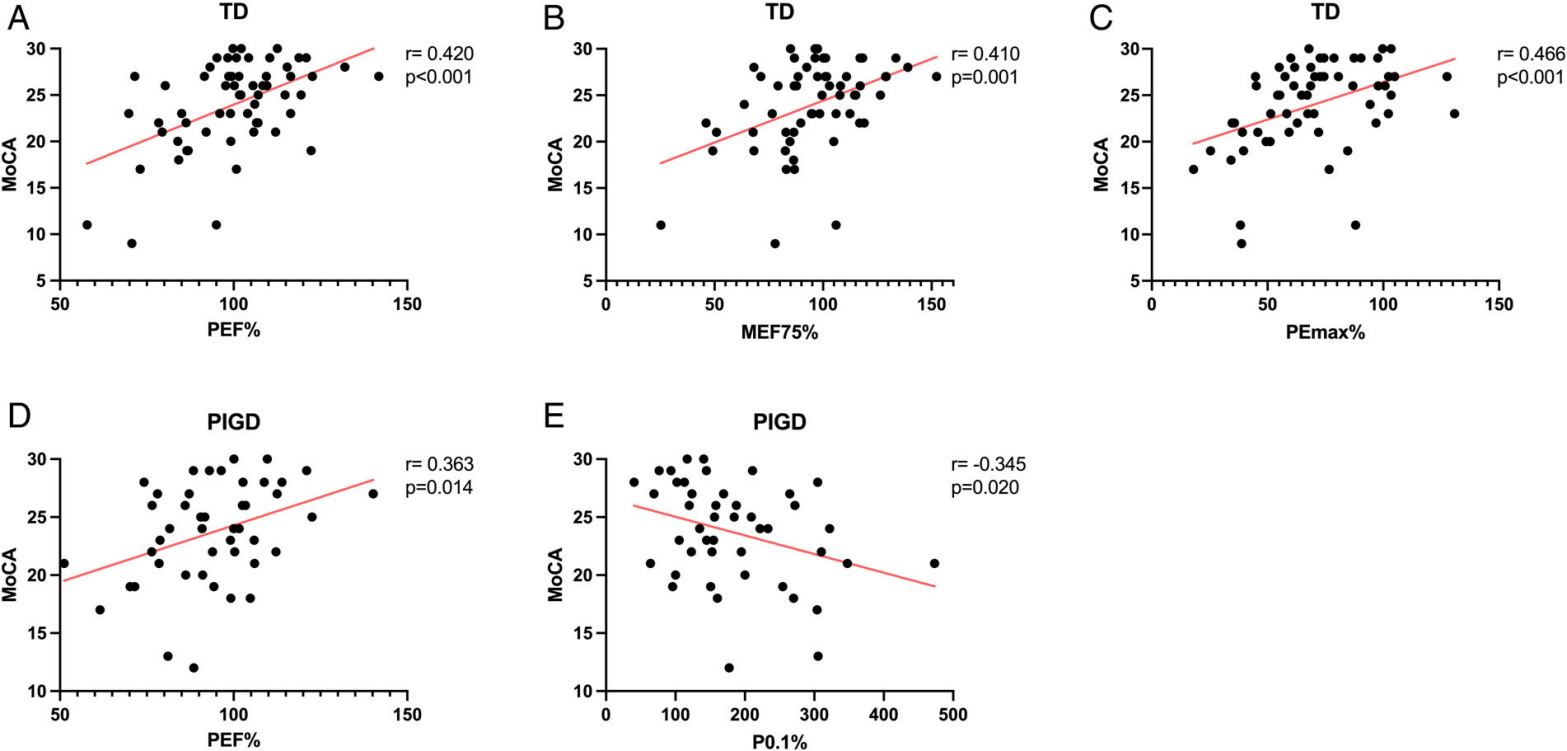
Correlation between respiratory function parameters and Montreal Cognitive Assessment (MoCA). Significant correlation (*P* < 0.05) is marked in solid red line.

Spearman correlation analyses were performed to explore the relationships between respiratory function parameters and clinical characteristics (Figs. 2 and 3). After being adjusted for potential confounders, a significant negative correlation was found between PImax% and MDS‐UPDRS III scores in the TD group (*r* = −0.319, *P* = 0.015, Fig. 2A). Similarly, in the PIGD group, PEF% and MEF75% were negatively correlated with MDS‐UPDRS III scores (PEF%: *r* = −0.343, *P* = 0.018; MEF75%: *r* = −0.333, *P* = 0.022, Fig. 2B, C).

Regarding cognitive function, our findings demonstrated positive correlations between FEP%, MEF75%, PEMAX%, and MoCA scores in TD patients (FEP%: *r* = 0.420, *P* < 0.001; MEF75%: *r* = 0.410, *P* = 0.001; PEMAX%: *r* = 0.466, *P* < 0.001; Fig. 3A–C). In the PIGD group, FEP% exhibited a significant upward trend with increasing MoCA scores (*r* = 0.363, *P* = 0.014; Fig. 3D). In contrast, higher P0.1% values were significantly associated with lower MoCA scores in PIGD patients, indicating a decline in cognitive function (*r* = −0.345, *P* = 0.020; Fig. 3E). No significant correlations were observed between respiratory function parameters and other clinical scales, including HAMA, HAMD, ESS, CCS, and PDSS‐2.

### Comparison of Respiratory Function Parameters in PD Patients with Different Stages of Cognition

Based on MoCA scores, patients in the TD and PIGD groups were further divided into three cognitive stages: PD with normal cognition (PD‐NC), mild cognitive impairment (PD‐MCI), and dementia (PD‐D). In the TD group, PEF%, MEF75%, and PEmax% progressively decreased with worsening cognitive function, with significant reductions observed in PD‐D compared to PD‐NC patients (PEF%: *P* = 0.009; MEF75%: *P* = 0.011; PEmax%: *P* = 0.002, Table 3). Although the differences in FEV1%, FVC%, TLC%, and PImax% across cognitive stages were not statistically significant, these parameters exhibited declining trends with cognitive deterioration. In the PIGD group, although no significant differences were detected across cognitive states, similar trends were observed, with declining PEF%, MEF75%, and PImax% values and increasing P0.1 values.

**TABLE 3:** Comparison of respiratory function parameters of Parkinson's disease (PD) motor subtypes in different cognitive stages of disease groups

	TD (n = 59)				PIGD (*n* = 47)			
Variables	PD‐NC (n = 27)	PD‐MCI (n = 21)	PD‐D (n = 11)	Group differences	PD‐NC (n = 18)	PD‐MCI (n = 18)	PD‐D (n = 11)	Group differences
Lung volume								
TLC%	90.80 ± 8.20	88.20 ± 11.55	85.99 ± 12.71	*P* = 0.615	88.37 ± 11.78	86.16 ± 8.24	87.97 ± 14.54	*P* = 0.672
RV%	91.47 ± 19.63	90.29 ± 18.83	94.60 ± 17.08	*P* = 0.785	96.49 ± 17.82	92.33 ± 16.21	107.04 ± 22.65	*P* = 0.172
FRC%	92.89 ± 26.66	92.42 ± 16.85	90.40 ± 21.32	*P* = 0.962	101.86 ± 19.13	90.73 ± 15.56	91.46 ± 22.13	*P* = 0.158
Spirometry								
FEV1%	104.10 ± 14.29	101.96 ± 16.53	92.35 ± 20.89	*P* = 0.251	95.68 ± 18.62	97.24 ± 12.04	96.00 ± 16.99	*P* = 0.884
FVC%	105.66 ± 15.90	104.12 ± 15.41	92.85 ± 19.80	*P* = 0.135	97.72 ± 16.99	97.94 ± 12.46	95.96 ± 13.44	*P* = 0.834
PEF%	105.27 ± 14.53	99.45 ± 13.30	87.31 ± 17.26	*P* = 0.012^b^	99.70 ± 16.90	92.91 ± 16.04	88.28 ± 17.33	*P* = 0.223
MEF25%	72.68 ± 41.69	70.20 ± 32.76	82.92 ± 42.18	*P* = 0.643	65.50 ± 25.93	79.20 ± 22.12	84.80 ± 30.30	*P* = 0.161
MEF50%	90.00 ± 29.79	84.01 ± 28.64	83.28 ± 27.95	*P* = 0.917	79.48 ± 23.96	82.59 ± 22.57	84.83 ± 33.08	*P* = 0.804
MEF75%	102.42 ± 20.00	94.03 ± 22.54	77.74 ± 23.35	*P* = 0.015^b^	96.28 ± 26.02	89.84 ± 25.66	80.04 ± 29.39	*P* = 0.386
Diffusion capacity								
DLCOc%	82.06 ± 15.87	84.32 ± 15.23	79.76 ± 13.90	*P* = 0.865	85.66 ± 17.16	78.43 ± 12.10	78.58 ± 14.28	*P* = 0.306
Respiratory muscle strength								
PImax%	39.56 ± 17.63	37.25 ± 13.72	28.15 ± 17.68	*P* = 0.113	31.02 ± 13.58	26.98 ± 13.92	21.27 ± 5.98	*P* = 0.113
PEmax%	79.22 ± 20.45	67.39 ± 25.06	49.46 ± 23.66	*P* = 0.002^b^	59.45 ± 19.33	52.65 ± 19.83	54.29 ± 21.41	*P* = 0.603
Respiratory center drive								
P0.1%	180.90 ± 94.44	138.41 ± 66.66	132.98 ± 66.13	*P* = 0.280	150.43 ± 73.30	201.81 ± 103.54	214.87 ± 86.88	*P* = 0.148

Abbreviations: TD, tremor‐dominant; PD‐NC, PD with normal cognition; PD‐MCI, PD with mild cognitive impairment; PD‐D, PD with dementia; PIGD, postural instability gait difficulty; TLC%, total lung capacity; RV%, residual volume; FRC%, functional residual capacity; FEV1%, forced expiratory volume in 1 second; FVC%, forced vital capacity; PEF%, peak expiratory flow; MEF25%, maximum expiratory flow rate at 25% of FVC exhalation; MEF50%, maximum expiratory flow at 50% of FVC; MEF75%, maximum expiratory flow at 75% of FVC; DLCOc%, diffusing capacity for carbon monoxide adjusted for hemoglobin; PImax%, maximal inspiratory pressure; PEmax%, maximal expiratory pressure; P0.1%, airway occlusion pressure at 100 milliseconds.

^a^ Differences were found between PDD and PD‐MCI.

^b^ Differences were found between PDD and PD‐NC.

^c^ Differences were found between PD‐MCI and PD‐NC.

### Multivariate Linear Regression for Cognitive Impairment in PD Motor Subtypes

Based on the correlation and subgroup analyses, the respiratory function parameters PEF%, MEF75%, PEmax%, and P0.1% were included as predictors in multivariate regression analyses. These parameters collectively explained 34.3% of the variance in MoCA scores for TD patients, with PEF% emerging as the most significant predictor (β = 0.497, *P* < 0.001, Table 4). In the PIGD group, PEF% and P0.1% together accounted for 13.1% of the variance in MoCA scores, with P0.1% identified as the primary contributing factor (β = −0.373, *P* = 0.015).

**TABLE 4:** Multiple linear regression analysis of Montreal Cognitive Assessment (MoCA) scores in tremor‐dominant (TD) and postural instability gait difficulty patients

	TD			PIGD		
Variables	β	*P*‐value	Adjusted *R* ^2^	β	*P*‐value	Adjusted *R* ^2^
PEF%	0.497	<0.001**	34.3%	0.089	0.548	13.1%
MEF75%	0.207	0.082				
PEmax%	0.239	0.050				
P0.1%				−0.373	0.015*	

Abbreviations: TD, tremor‐dominant; PIGD, postural instability gait difficulty; PEF%, peak expiratory flow; MEF75%, maximum expiratory flow at 75% of FVC; PEmax%, maximal expiratory pressure; P0.1%, airway occlusion pressure at 100 milliseconds.

** *P* < 0.001.

* *P* < 0.05.

## Discussion

This study aimed to evaluate respiratory function differences in PD motor subtypes and identify potential markers for disease progression. The key findings can be summarized as follows: (1) Both TD and PIGD patients exhibited impairments in pulmonary ventilation, respiratory muscle strength, and respiratory drive, whereas respiratory function impairments were more severe in PIGD patients. (2) P0.1 demonstrated high performance in distinguishing PD motor subtypes from HCs; however, respiratory function parameters showed limited utility in differentiating PD motor subtypes. (3) Respiratory function indices might serve as markers of motor severity and cognitive function in different PD motor subtypes. In TD patients, respiratory dysfunction worsened with cognitive decline, with PEF% identified as a significant determinant of cognitive status. Overall, these findings illustrate that PIGD patients experience more severe respiratory impairments, and respiratory function indices may serve as auxiliary markers for motor and cognitive function in PD motor subtypes.

Accumulating evidence has highlighted varying degrees of respiratory dysfunction in PD patients. In terms of ventilation, prior cohort studies reported significantly reduced PEF in PD patients compared to HCs, with improvements following levodopa treatment, suggesting that PEF abnormalities might result from respiratory muscle discoordination and upper airway obstruction.^15^, ^16^, ^17^ Consistent with these findings, we observed reduced PEF% in both TD and PIGD patients compared to controls. However, in contrast to previous findings indicating significant declines in FEV1 and FVC in PD patients,^6^ our data demonstrated reduced FVC% and TLC% only in PIGD patients, possibly due to progressive respiratory muscle weakness. PIGD patients are generally older, experience faster disease progression, exhibit higher rates of cognitive impairment, and present with more severe axial symptoms, such as rigidity and freezing.^4^, ^18^, ^19^ In line with these clinical features, the present study further demonstrated significantly lower PImax% and PEmax% in PIGD patients compared to TD patients, potentially attributable to reduced chest wall compliance, severe respiratory muscle dysfunction, and rigidity.

P0.1, defined as the airway pressure at 100 milliseconds after the onset of inspiratory effort in a closed circuit,^20^, ^21^ remains unaffected by airflow during measurement, making it a robust indicator of respiratory drive.^22^ Our previous study demonstrated significantly elevated P0.1% in early idiopathic PD patients compared to HCs, underscoring its diagnostic potential.^7^ Consistent with these findings, we observed increased P0.1% in PD patients, likely due to heightened respiratory load resulting from airway resistance and reduced lung compliance. Although P0.1% exhibited strong diagnostic value in distinguishing PD patients from HCs, it was not particularly effective in differentiating motor subtypes. Overall, both TD and PIGD patients displayed varying degrees of impaired ventilation, respiratory muscle strength, and respiratory drive, with PIGD patients experiencing more severe impairments.

Motor symptoms, including bradykinesia, rigidity, tremor, and postural abnormalities, are hallmark manifestations of PD. However, the clinical spectrum also encompasses non‐motor features, such as cognitive decline, depression, constipation, and sleep disturbances, which substantially contribute to disability.^23^, ^24^ Despite different PD motor subtypes exhibit distinct clinical characteristics and progression trajectories, reliable markers for subtype‐specific disease progression remain limited. Previous studies have reported significant negative correlations between respiratory function parameters (eg, PImax, PEmax) and disease severity in PD patients,^25^ as well as associations between FVC, FEV1, and MDS‐UPDRS III scores.^15^ Our findings corroborated these results, showing correlations between respiratory function indices and motor severity in different PD subtypes. For example, PImax% was negatively correlated with MDS‐UPDRS III scores in TD patients, whereas PEF% and MEF75% were inversely associated with motor severity in PIGD patients.

Epidemiological studies indicate that the prevalence of cognitive impairment in PD patients is six times higher than that in healthy populations, representing one of the most prominent non‐motor manifestations of PD.^26^, ^27^ Generally, PIGD patients have a higher prevalence of cognitive impairment compared to TD patients, but no significant differences in overall cognitive scores were observed between TD and PIGD patients in this study. Poor baseline respiratory function predicts faster cognitive decline in memory, temporal orientation, and executive functions.^28^, ^29^ Recent studies have shown that impaired respiratory function increases the risk of progression from mild cognitive impairment to dementia, possibly through mechanisms involving accelerated brain atrophy and microvascular damage.^10^ Our findings indicated positive correlations between PEF%, MEF75%, PEmax%, and MoCA scores in TD patients. Conversely, increased P0.1 was associated with cognitive decline in PIGD patients. Further subgroup analyses substantiated that PEF%, MEF75%, and PEmax% progressively declined with worsening cognitive status in TD patients. Multivariate linear regression confirmed that PEF% significantly influenced cognitive status in TD patients, whereas P0.1% was a determinant in PIGD patients. However, the limited explanatory power of the regression model for cognitive function in PIGD patients highlights the complexity of these interactions and warrants further investigation. The mechanisms underlying the relationship between respiratory dysfunction and cognitive impairment remain unclear. Chronic hypoxia and inflammatory responses associated with respiratory dysfunction may adversely affect neurotransmitter synthesis, oxidative stress, and blood–brain barrier integrity.^30^ Cognitive impairment in PD involves changes in central neurotransmitter systems,^31^, ^32^ inflammation, oxidative stress, and pathological protein aggregation (eg, β‐amyloid and tau deposition).^33^, ^34^ Future imaging and pathophysiological studies are needed to elucidate these mechanisms and to determine whether respiratory dysfunction contributes to the progression from prodromal dementia to clinical dementia in PD.

This study has several strengths, including the novel comparative analysis of respiratory function impairments in PD motor subtypes and the identification of potential markers for disease progression. Nonetheless, there are limitations worth to be mentioned. First, the study lacks longitudinal data, deficient in an assessment of dynamic changes in respiratory function and cognitive status over time. Second, although major confounders were adjusted for, the influence of residual or unmeasured confounding cannot be excluded. Moreover, cognitive assessments were not conducted in HCs, restricting comparisons between respiratory‐cognitive interactions in PD patients and the general aging population.

In conclusion, PIGD patients exhibit more severe respiratory impairments than TD patients, and respiratory function indices may serve as auxiliary markers for disease progression in different PD motor subtypes. Future multicenter, longitudinal studies are needed to confirm these findings and explore the underlying neuropathological mechanisms.

## Author Roles

(1) Research project: A. Conception, B. Organization, C. Execution; (2) Statistical analysis: A. Design, B. Execution, C. Review and critique; (3)Manuscript preparation: A. Writing of the first draft, B. Review and critique;

Z.L.: 1A, 1C, 2A, 2B, 2C, 3A

T.L.H.: 1C, 2B, 3A

N.Z.: 1A, 2A, 2C, 3B

X.S.: 1C, 2B, 3A

Y.L.W.: 1C, 2B, 3A

P.C.H.: 1C, 2B, 3A

Q.Q.S.: 1C, 2B, 3A

T.Y.C.: 1C, 2B, 3A

T.B.Z.: 1C, 2B, 3A

H.R.W.: 1C, 2B, 3A

X.D.Z.: 1A, 1B, 2C, 3B

## Disclosures

**Ethical Compliance Statement:** The study was approved and performed in accordance with the guidelines of the Ethics Committee of Tianjin Medical University General Hospital (reference number IR2017‐168‐01). All participants signed a written informed consent form before being recruited for the study. We confirm that we have read the journal's position on issues involved in ethical publication and affirm that this work is consistent with those guidelines.

**Funding Sources and Conflict of Interest:** This study was supported by grants from National Natural Science Foundation of China (grant number: 82471276) and Natural Science Foundation of Tianjin Municipality (grant number: 23JCYBJC00690). The authors disclose no financial and personal relationships with other people or organizations that could inappropriately influence this study.

**Financial Disclosures for the previous 12 months:** The authors declare that there are no additional disclosures to report.

## Data Availability

The data that support the findings of this study are available from the corresponding author upon reasonable request.
